# Back to Natural Fiber: Wool Color Influences Its Sensitivity to Enzymatic Treatment

**DOI:** 10.1100/2012/356239

**Published:** 2012-04-26

**Authors:** Amro A. Amara

**Affiliations:** ^1^Protein Research Department, Genetic Engineering and Biotechnology Research Institute, Mubarak City for Scientific Research and Technology Applications, Alexandria, Egypt; ^2^Microbiology Division, Pharmaceutics Department, College of Pharmacy, King Saud University, P.O. Box 2457, Riyadh 11451, Saudi Arabia

## Abstract

There are many missed biotechnological opportunities in the developmental countries. Wool quality improvement is one of them. This study is concerning with improving the wool quality using technical enzymes. White wool proves to be more susceptible to the enzymatic treatment than blackish brown wool. This proves that the enzymatic reaction is sensitive to the natural color differences between wool fibers. A simple enzymatic method has been used to improve the wool quality as well as to investigate the changes happened in the wool fibers. *Geobacillus stearothermophilus* has been used under mesophilic and static cultivation conditions using wool as the main carbon source. These conditions prove to be more suitable for maintaining the fiber structure, less expensive, and reliable as an in-house biotechnological process that can be adapted everywhere. The enzyme activity in case of white wool was 4 Units/ml and for blackish brown wool was 1.5 Units/ml. Electron microscope has been used to evaluate the end result. By following the process included in this paper using probable microbial strain(s), the wool quality improvement can be applied globally and can add another value to the economy of the developmental countries.

## 1. Introduction

Salem et al. [1] show the possibility to produce thermophilic proteases enzymes under mesophilic conditions [1]. This will add a significant possibility to use low-price technology in different technical enzymes' applications. Amara and Serour show the possibility to improve the wool quality using *Bacillus sp.*; however, they use thermophilic conditions [2]. Additionally Amara et al. introduce the idea of using proteases and lipases as biodetergents which can stand alone without the use of additional chemicals constituents [3]. Enzymes are a renewable source which can be implemented in different industrial and technical applications including paper industry, detergent, drugs, degradation of different wastes, textile, food, pharmaceutical, and leather [2–9]. Thermostable enzymes are active and stable at temperature higher than optimal growth of their producer strains [3, 10]. Relative ease of isolation of Bacilli from diverse sources has made these organisms the focus of attention in biotechnology [11]. Wool is a fabric made from the hair of sheep; being one of the oldest textile fibers known, it has unique properties and considered as being a masterpiece of design [12]. Wool absorbs moisture vapor, resulting in superior comfort in both hot and cold weathers [12]. Wool has the ability to insulate against heat and cold, which protects body against sudden change of temperature [12]. Due to their excellent properties, nowadays wool market counts by billions of dollars and the economy of some countries depends so far on the profits of exporting wool either as crude or as a textile products [4, 12]. Its complex cellular structure also enables it to absorb moisture vapor but repel liquid-try and soak up water with wool clothe. No synthetic fibers have been able to combine all these characteristics [12]. “Facing up” is the trade term for the ruffling up of the surface of wool garments by abrasive action during dyeing. Enzymatic treatment reduces facing up which significantly improves the pilling performance of garments and increases softness [13–18]. Other contributors to environmental pollution are the finishing processes of wool that involve oxidation of the wool surface by means of chlorination or application of softening agents to modify the handle and improve these properties. Hand and dyeing behaviors are important quality aspects for wool [19]. These processes have disadvantages as they are sources of environmental pollution. For this reason, attention of tanners is focused towards revamping the processing methods, recovery system, and effluent treatment techniques in order to develop environmental friendly alternative processes and to make processing eco-friendly. Enzymes can be used as an alternative technology for pretanning processes [19]. Enzymatic treatments could improve the wool quality as proved in this study.

## 2. Material and Methods

### 2.1. Bacillus Strains and Growth Conditions

*Geobacillus stearothermophilus* strain has been used in this study. It shows an ability to grow statically in wool and tap water at temperature from 25 ± 5°C. It grows routinely in LB medium (Luria-Bertani) [20] at 37°C and maintained at −70°C by adding 300 *μ*L glycerol to each 1 mL culture in suitable plastic container.

### 2.2. Preparation of L-Tyrosine Standard Curve

1.1 mM L-tyrosine was dissolved in 100 mL deionized water by heating gently (without boiling). After complete dissolving of the L-tyrosine, the standard curve generated by reading the absorbency for 0, 12.5, 25, 50, 100, 200, 250, and 500 *μ*L from L-tyrosine solution completed to 1 mL by adding deionized water at 280 nm. The relationship between the absorbency and the mM L-tyrosine then was plotted as y/x line plot.

### 2.3. Preparation of Casein-Universal Buffer for pH 9 Enzyme Activity

Universal buffer was prepared according to Britton and Robinson [21] which consists of 40 mM H_3_PO_4_, 40 mM acetic acid and 40 mM H_3_BO_3_. The buffer pH was adjusted to 9 using 0.2 M NaOH. 0.325 mg casein weighted and dissolved in 50 mL of Universal buffer (pH 9). The mixture dissolved by heating gently to 80–90°C without boiling. The mixture used immediately or incubated for short time at 4°C [not longer than 12 hr].

### 2.4. Static Wool Quality Improvement Experiment

Crude white and blackish brown wool fibers have been collected from the Egyptian company STIA (before any treatment). This wool was not used in the production of clothes due to its unsuitable quality. However, it is used in carpet and low quality sleeping covers. Each type of wool has been washed (in lab) using tap water to remove all dirt. Then the wool has been dried at room temperature (about 25°C). One gram of each type of wool fibers has been added to 250 mL flasks and autoclaved for 5 min at 121 par. A 5 mL LB medium in test tube has been used to prepare fresh culture (OD about 0.8 nm) of *Geobacillus stearothermophilus* using shaker incubator (125 rpm). 25 *μ*L from the previous culture has been added to each flask. The flasks were kept in room temperature for one week. At the end of the process, samples have been collected for enzyme activity and fibers have been collected for electron microscope examination.

### 2.5. Enzyme Activity

300 *μ*L of each supernatant which contain the enzyme has been added to the same volume of the Casein-Universal buffer solution (pH 7). The enzymes-substrate mixture then was incubated at 25°C for 30 min. After the incubation period, the enzyme reaction stopped by adding 600 *μ*L of 10% trichloroacetic acid. The mixture allowed to stand at room temperature for 15 min then centrifuged at 10000 rpm for 10 min (Biofuge 15-Heraeus Sepatech). The absorbance of each sample determined spectrophotometrically at 280 nm (PerkinElmer-.UV/VIS Spectrometer Lambda) and their tyrosine content derived from the tyrosine standard curve and the enzyme activity determined as Units/mL.

### 2.6. Sample Preparation for Electron Microscopy

To study the enzymatic activities which improve the wool surface, electron microscope was used to scan the surface of the treated and untreated wool. The samples include treated and untreated white and blackish brown fibers as well as a Marino fiber. A single fiber from each of the treated and untreated wool fibers has been fixed in the surface of flat glass slid and washed gently by distilled water for 3 sec then allowed to dry at 37°C. The surface of the dry wool fiber then coated with approximately 15 nm gold (SPI-Module TM sputter Coater).

### 2.7. Scanning of the Wool Surface

The golden coated sample then subjected to be scanned by analytical scanning electron microscope (Jeal JSM-6360LA) with secondary element at 20 KV acceleration voltages at room temperature. The digital images were adjusted and saved.

## 3. Results and Discussion

The opportunity to use direct and in-house method for wool quality improvement will support the life of many people all over the world. This will be done particularly if a simple technology has been used. In fact, wool quality improvement is a missed part of the economy of the developmental countries which are in demand for any kind of support to improve their lives. In a previous study done by Salem et al. the possibility of producing thermophilic enzymes under mesophilic conditions open the way for many new ideas in the fields of industrial and technical applications [1].

**Figure 1:**
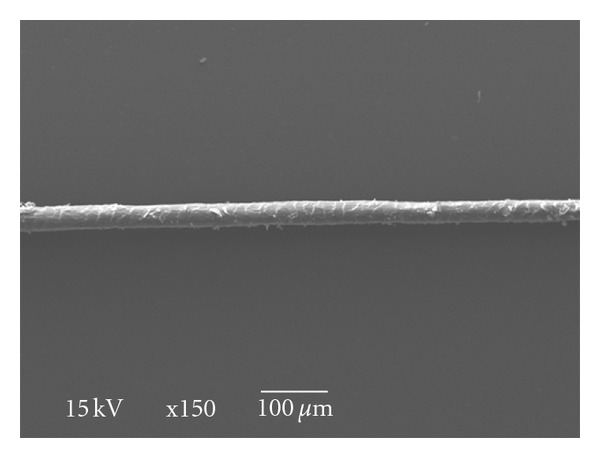
Marino wool fiber (×150).

**Figure 2:**
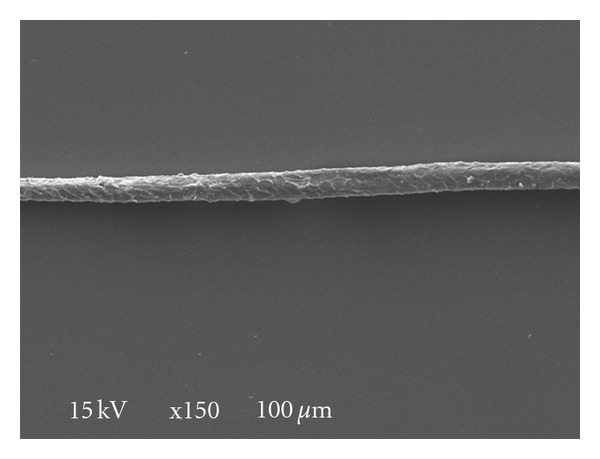
Untreated Egyptian white wool fiber (×150).

**Figure 3:**
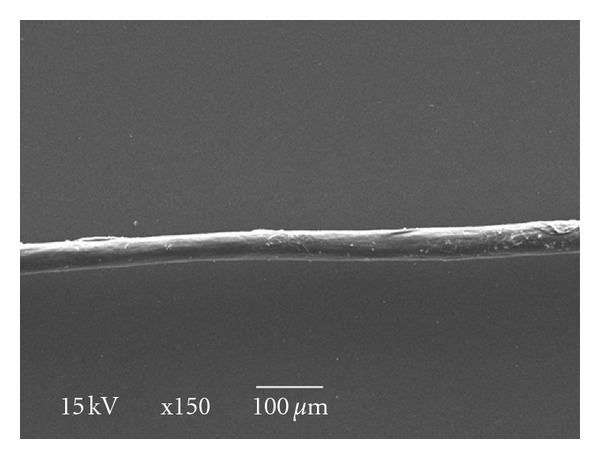
Treated Egyptian white wool fiber (×150).

**Figure 4:**
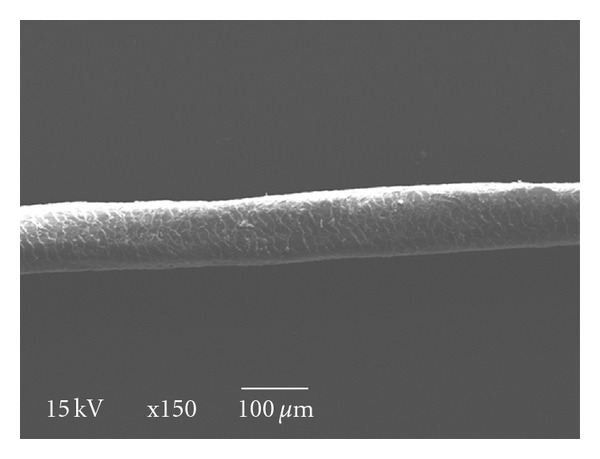
Untreated blackish brown wool fiber (×150).

**Figure 5:**
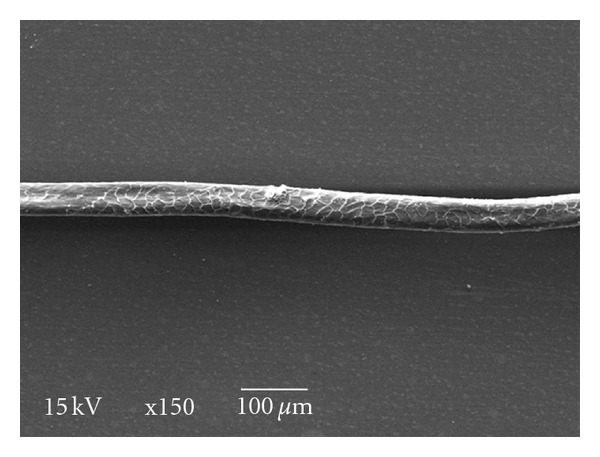
Treated blackish brown wool fiber (×150).

This concept can be applied also in the production of enzymes which can be used in wool fiber quality improvement. Wool quality-improving enzymes can turn to be wool degrading enzymes if the activities of the used enzymes were not reduced to the level which did not harm the fiber. I have been observing that the most technical and critical point is the temperature which can inhibit the enzyme activity tremendously. Reducing the temperature will be our start point to use the enzymes to improve the wool quality rather than induce fiber degradation. The process has been adjusted to be reliable, inexpensive, and in-house. Tap water has been used as a medium for treatment process. Amara and Serour [2] recommended using tap water only, where it can supply the Bacillus strain with its requirement from trace elements to start the process. The impurities, which still contaminate the wool fibers, will be able to cover any shortage and will allow Bacillus to adapt itself. Amara and Salem show that bacterial strains will not use the target carbon source if there is another simpler one existing in the medium [22]. Wool has been used (only) as a carbon source. White and blackish brown wool has been used in this study. The white wool proves to be more sensitive to the process with enzymatic activity of about 4 Units/mL, while for blackish brown wool it was about 1.5 Units/mL. The biological system is sensitive to the natural differences between the wool fibers. A simple method has been used to control the performance on the wool fiber surface quality improvement. Authentic sample of the Marino fiber has been examined also (without treatment). The surface of the white wool has been perfectly improved while that of the blackish brown wool has been slightly improved (comparing with untreated wool) as in Figures 1, 2, 3, 4, and 5. The protocols used in this study have been simplified to be reliable, convenient, inexpensive, and in-house global protocols for wool quality improvement. Keratinase and other proteolytic enzymes produced from wool quality improvement processes can be considered as an additional value in many biotechnological applications. The produced enzymes can be used alone in many applications in chemical-free formula as described by Amara et al. [3, 22], or in different technical enzymes applications. Wool quality improvement can improve the genetically poor quality wool fibers, and color is an important factor that must be considered during the treatment process.
